# Phenotypic variability of *GABRA1*‐related epilepsy in monozygotic twins

**DOI:** 10.1002/acn3.50895

**Published:** 2019-09-30

**Authors:** Martin Krenn, Margot Ernst, Matthias Tomschik, Marco Treven, Matias Wagner, Dominik S. Westphal, Thomas Meitinger, Ekaterina Pataraia, Fritz Zimprich, Susanne Aull‐Watschinger

**Affiliations:** ^1^ Department of Neurology Medical University of Vienna Vienna Austria; ^2^ Institute of Human Genetics Technical University Munich Munich Germany; ^3^ Center for Brain Research Medical University of Vienna Vienna Austria; ^4^ Institute of Human Genetics Helmholtz Zentrum München Neuherberg Germany; ^5^ Institute of Neurogenomics Helmholtz Zentrum München Neuherberg Germany

## Abstract

Variants in *GABRA1* have been associated with different epilepsies ranging from mild generalized forms to epileptic encephalopathies. Despite the broad clinical spectrum, phenotypes were found to be largely concordant within families. Contrary to this observation, we report monozygotic twin sisters with generalized epilepsy due to the c.541C>T; p.(Pro181Ser) *de novo* variant in *GABRA1*. One experienced juvenile absence seizures promptly responding to first‐line medication, whereas the second developed severe treatment‐refractory epilepsy with febrile, absence, atonic, and tonic‐clonic seizures indicating marked intrafamilial variability in *GABRA1*‐related epilepsy. Moreover, we provide a molecular characterization of the novel variant based on recently published structural data.

## Introduction

Generalized epilepsies are clinically characterized by absence, myoclonic or tonic‐clonic seizures and an electroencephalographic pattern of generalized polyspikes or spike‐and‐wave discharges.^1^ Although high concordance rates in twin studies support a substantial genetic background, the underlying molecular architecture is complex and largely elusive.^2^ The majority of cases is considered to be polygenic with both common and rare genetic variants contributing to disease causation.^3^, ^4^, ^5^ However, also rare monogenic (dominantly inherited or *de novo*) mutations have been reported. Despite the broad molecular spectrum, the underlying biological pathways are often shared between complex and monogenic forms, with γ‐aminobutyric acid type A (GABA_A_) receptor genes playing a major role.^6^ Among others, mutations in *GABRA1*, encoding the α1 subunit of the GABA_A_ receptor, have been identified in juvenile myoclonic epilepsy (JME) and childhood absence epilepsy (CAE).^7^, ^8^ With the increasing number of reported families, the spectrum ranges from these mild subtypes to severe developmental and epileptic encephalopathies (DEE).^9^, ^10^ Although clinically heterogeneous, it has been emphasized that previously reported phenotypes were largely concordant among mutation carriers within the same family (e.g., as opposed to *SCN1A*).^11^

In contrast to this notion, we report monozygotic twin sisters with generalized epilepsy and developmental delay carrying the same (previously undescribed) *de novo* variant in *GABRA1*, but clinically presenting with remarkable differences regarding disease severity and response to antiepileptic drugs (AED).

## Patients and Methods

### Probands and samples

All subjects gave written informed consent for the collection and storage of clinical data, blood samples, experimental analyses, and publication. The study was conducted in agreement with the Declaration of Helsinki and approved by the local ethics committee.

### Exome sequencing

Exome sequencing (ES) was performed for both sisters using *SureSelect Human All Exon Kit* 60 Mb, V6 (Agilent, Santa Clara, California, USA) for exome enrichment. Libraries were sequenced on an *Illumina HiSeq4000* system (Illumina, San Diego, California, USA).^12^ Reads were aligned to the UCSC human reference assembly (hg19). More than 98% of the exome were covered at least 20‐fold. Average coverage was more than 125‐fold. Variant prioritization was performed based on autosomal recessive (minor allele frequency, MAF < 0.1%) and autosomal dominant (MAF < 0.01%) filters. For variant interpretation, the standard criteria of the American College of Medical Genetics and Genomics (ACMG) were applied.^13^

### Structural analysis

The recently released 3D structures of α1‐containing GABA_A_ receptors (6HUP, 6D6T) were evaluated and visualized with MOE.^14^, ^15^ The subdomain of interest overlaps well in these structures. 6HUP was used for further analysis. Sequences were obtained from SwissProt and aligned with ClustalX.

## Results

### Clinical findings

#### Patient I

This female patient was the first‐born of two monozygotic twin sisters of neurologically healthy, nonconsanguineous parents. Pregnancy and birth were unremarkable (vaginal delivery). She was born at term with a weight of 2650 g, a body length of 49 cm and a head circumference of 35 cm (subsequently 49 cm at 9 and 21 months of age). Mild to moderate developmental delay was noted early on. She was able to crawl at 12 months, learned to walk unaided at 19 months and started to speak at 2 years of age. After attending elementary school, she changed to a special‐needs school.

**Figure 1:**
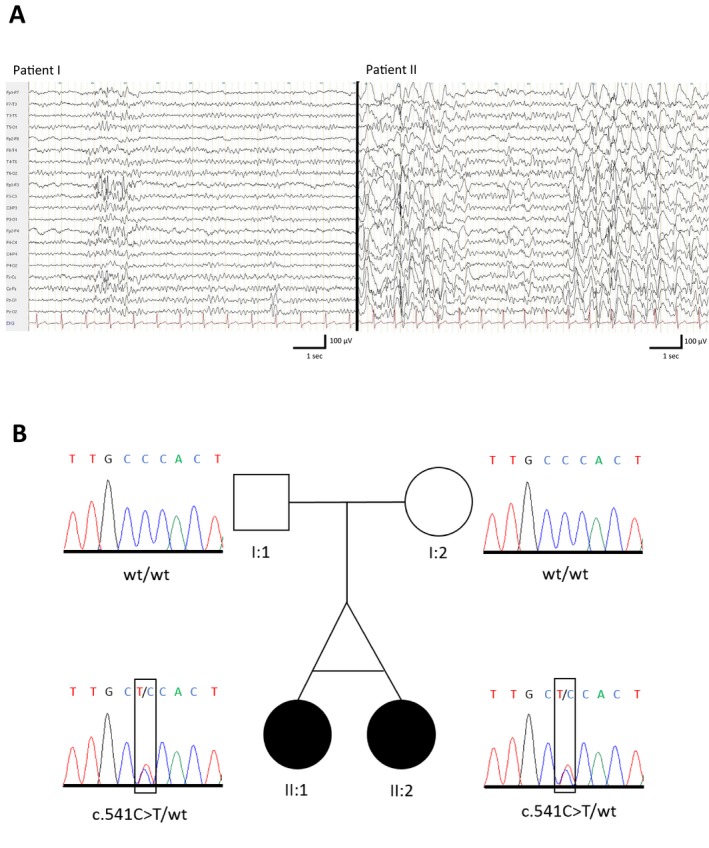
Illustrations of EEG recordings and family pedigree. (A) Comparison of the EEG recordings between the two sisters showing the rare occurrence of generalized polyspikes (with left frontal maximum) lasting for 1 second in the mildly affected sister (Patient I); and epileptic discharges with generalized (poly)spike‐wave activity (partly lasting up to 2 min) in the severely affected sister (Patient II). (B) Pedigree illustration and electropherograms for the *de novo* c.541C> T; p.(Pro181Ser) variant that could be confirmed by Sanger sequencing in both monozygotic twin sisters, but not in the parents (pathogenic variant framed)

At the age of 12 years, absence seizures were noted for the first time. There was no reported history of other seizure types at any time. Within one year after the onset of absence seizures, an AED trial with lamotrigine (400 mg daily) and add‐on treatment with levetiracetam (2000 mg daily) was initiated. Routine EEG recordings showed rare generalized polyspikes (Fig. 1A). Aged 25 years now, the patient has been completely seizure‐free for more than 13 years without any medication‐related side effects. At the last follow‐up, she was employed at a sheltered workshop.

#### Patient II

Patient II was born 20 min after her twin sister (breech presentation, subsequent vaginal delivery) with a weight of 2370 g, a body length of 47 cm and a head circumference of 33 cm (47 cm at 9 months, 49 cm at 21 months of age). Similarly, psychomotor and language delay was noted after birth, but she started to speak simple words only at the age of 2.5 years. According to a neuropsychological assessment, developmental delay had been slightly more pronounced in Patient II.

In contrast to her sister, she also suffered two febrile seizures during her first year of life. Onset of absence seizures was at the age of 8 years and she occasionally also displayed atonic seizures (loss of muscle tone and subsequent fall). Additionally, she experienced bilateral tonic‐clonic seizures. From 8 to 21 years of age, she was seizure‐free under a monotherapy with lamotrigine. However, thereafter, both absence and bilateral tonic‐clonic seizures recurred leading to severe treatment‐refractory epilepsy. Multiple AED trials including lamotrigine (500 mg daily), levetiracetam (1500 mg daily), perampanel (8 mg daily), topiramate (100 mg daily), clobazam (15 mg daily), ethosuximide (500 mg daily), and valproic acid (2500 mg daily) either as monotherapy or in combination did not result in seizure control. The current AED regimen includes lamotrigine (150 mg daily), valproic acid (1000 mg daily) and perampanel (6 mg daily). On average, she still experiences five absence seizures per day and up to five bilateral tonic‐clonic seizures per month. Recent video‐EEG monitoring revealed interictal 2.5Hz spike waves lasting up to 2 min and subclinical epileptiform activity with generalized polyspike waves during sleep. Recorded absence seizures were accompanied by generalized polyspikes and 2.5‐3Hz spike wave activity (Fig. 1A).

Brain MRI (Figure S1) and neurological examination did not reveal any abnormalities in the two patients. Phenotypic details are summarized in Table 1.

**Table 1:** Main phenotypic characteristics of the monozygotic twin sisters reported in this study.

	Patient I	Patient II
Gender	Female	Female
Current age	25 years (2019)	25 years (2019)
Age of (afebrile) seizure onset	12 years	8 years
Febrile seizures	No	Yes (2 FS during first year of life)
Seizure types	Only absence seizures	Absence seizures, atonic seizures, bilateral tonic‐clonic seizures
EEG	Rare generalized polyspikes (1 sec)	Clinical and subclinical epileptiform activity with 2.5Hz spike waves and polyspikes lasting up to 2 min
Intellectual disability	Mild to moderate	Moderate
AED trials	LTG, LEV	LTG, VPA, LEV, PER, CLB, ESM, TPM
AED response	Completely seizure‐free under LTG and LEV for 13 years	Severe AED resistance with daily seizures under various AED combinations
MRI brain	Normal	Normal

AED, antiepileptic drugs, CLB, clobazam, EEG, electroencephalography, ESM, ethosuximide, FS, febrile seizures, LEV, levetiracetam, LTG, lamotrigine, MRI, magnetic resonance imaging, PER, perampanel, TPM, topiramate, VPA, valproic acid

### Molecular findings

ES identified the heterozygous missense variant NM_000806.5: c.541C>T; p.(Pro181Ser) in *GABRA1* in both patients. The variant was absent from the Genome Aggregation Database (gnomAD) and our in‐house database (>18,000 exomes). In silico tools consistently predicted the variant to be deleterious (PolyPhen‐2: 0.942, SIFT: 0.04, CADD: 25.9, M‐CAP: 0.179). The variant was not detected in either parent using Sanger sequencing, suggesting *de novo* mutagenesis (Fig. 1B). According to ACMG criteria, the variant was classified as pathogenic.

**Figure 2:**
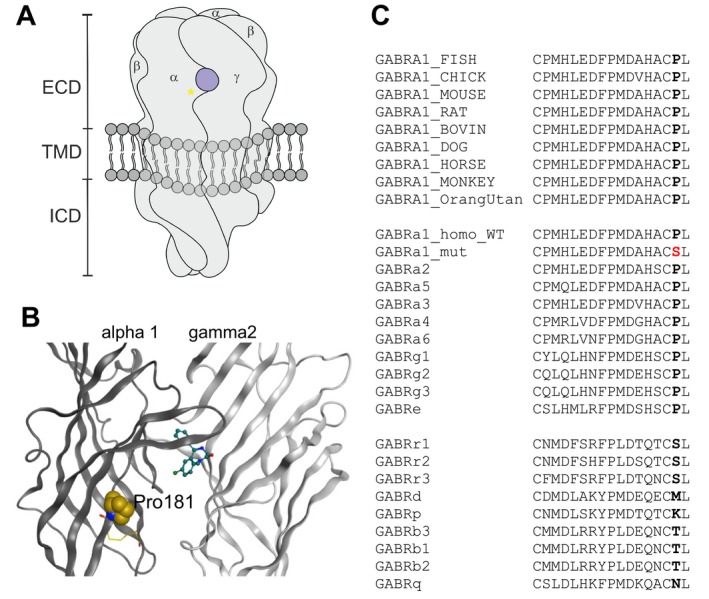
(A) Schematic view of a pentameric GABA_A_ receptor with the benzodiazepine‐binding site in view. The yellow asterisk indicates the relative localization of the mutation. (B) Rendering of the extracellular domain of the alpha1 and gamma2 subunits in the 6HUP structure in complex with diazepam. The cys‐loop forming cysteines are rendered as yellow sticks, the Pro181 (protein numbering: Pro154) is rendered in space filling representation, diazepam is rendered in stick representation. (C) Partial alignment showing the cys‐loop and the two aa C‐terminal of it. Top group: GABRA1 sequences for representative species; middle group: human subunit sequences for the alpha‐like family branch of GABA_A_R subunits – the mutation is shown in red; bottom group: human subunit sequences for the beta‐like family branch. The Pro is seen to be conserved among the alpha‐like family branch, and highly conserved in GABRA1 in the animal kingdom.

Computational analysis of the available structures revealed that the p.(Pro181Ser) missense variant is localized just C‐terminal of the cys‐loop of the α1‐subunit (Fig. 2). This is a highly conserved subdomain that is known to couple conformational changes between the ligand‐binding domain and the channel domain.^16^ The point mutation is localized in close proximity to the benzodiazepine‐binding site.

## Discussion

*GABRA1*‐related epileptic disorders have been shown to encompass a broad clinical spectrum spanning from mild generalized epilepsies to severe infantile DEE. However, marked phenotype variability within families (as in *SCN1A*‐related disorders) has not been reported thus far.^11^ Demonstrating variable intrafamilial expression, we describe monozygotic twin sisters with *GABRA1*‐related generalized epilepsy and striking phenotypic differences regarding severity and treatment response.

While the degree of intellectual impairment is comparable between the sisters, seizure types and the clinical disease course differ remarkably. First, only the more severely affected sister had infantile febrile seizures, bilateral‐tonic‐clonic seizures and atonic seizures (which are all part of the already known *GABRA1* spectrum). Moreover, the onset of absence seizure, which is the only seizure type shared between the sisters, was 4 years earlier. Most importantly, the benign course in Patient I, with absence seizures immediately responding to first‐line medication, is opposed to severe treatment resistance with a high seizure frequency despite multiple AED trials in Patient II.

The cause of this variable phenotypic expression remains open to speculation. The identical genetic background of monozygotic twins allows to exclude potentially contributing germline background variation such as additional common variants of low or intermediate effect sizes. Theoretically, the differences could be explained by unidentifiable genetic factors such as later developing somatic mutations or by environmental factors. Second, we acknowledge that it cannot be excluded that the mildly affected sister may develop treatment‐refractory epilepsy or different seizure types later in the course, so that the differences could actually be less pronounced than it presently appears.

In previously described subjects with *GABRA1*‐related epilepsy, the clinical course varied depending on the underlying mutation and the observed mode of inheritance. Generally speaking, *de novo* variants have been associated with more severe phenotypes and infantile seizure onset (often leading to DEE).^11^ In contrast, our reported patients had comparably late seizure onset in spite of harboring a *de novo* variant and do not fulfill the diagnostic criteria for DEE. Although the variant was not detected in blood DNA of the parents, parental mosaicism (i.e., *de novo* mutagenesis already occurring in parental tissue) – an increasingly appreciated phenomenon in epilepsies – cannot be excluded in this family.^17^

Since the variant reported here has not been described previously, we performed a structural analysis to draw conclusions on the possible change in function resulting from the variant. The high degree of conservation among both orthologs and paralogs suggests conserved function of this subdomain, which has been shown to couple ligand‐binding events to the channel domain (Fig. 2).^16^ Thus, the structural analysis suggests that domain coupling and responses to endozepines or benzodiazepine drugs may be impaired.^18^ Further experimental testing would be required to reveal whether molecular assembly, trafficking of the pentamer, benzodiazepine binding and action, or GABA responses are affected.

Taken together, our report suggests that significant intrafamilial phenotypic variability can be observed in *GABRA1*‐related seizure disorders. Furthermore, it supports considering pathogenic *de novo* variants in syndromic (generalized) epilepsies also with noninfantile seizure onset.

## Author Contribution

MK drafted the manuscript and was involved in genetic data analysis. SAW proposed and supervised the manuscript and was involved in the clinical management of both patients. MW, DSW, and TM contributed to the interpretation of genetic data. FZ, MTo, MTr, and EP contributed to clinical data acquisition. ME performed computational structure and sequence analysis. All authors read and approved the manuscript before submission.

## Conflict of Interest

The authors declare that they have no conflict of interest related to the content of this article.

## Supporting information


**Figure S1**. Brain MRI of the more severely affected sister (Patient II). (A) Coronal and (B) axial T2‐weighted Fluid Attenuated Inversion Recovery (FLAIR) sequence without any structural (epileptogenic) abnormalities.
