# Supplementary figures and images for: Phenotypic variability of GABRA1‐related epilepsy in monozygotic twins
Source: Ann Clin Transl Neurol. 2019 Sep 30;6(11):2317–22. doi: 10.1002/acn3.50895 (PMC6856628; doi:10.1002/acn3.50895)

A

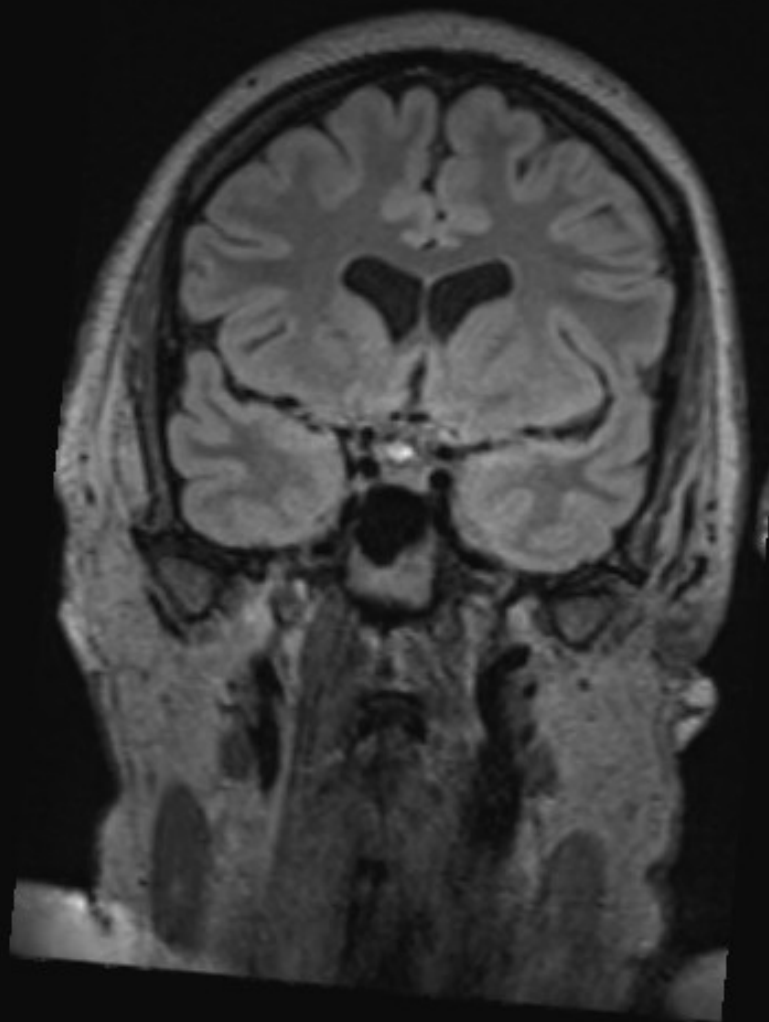

B

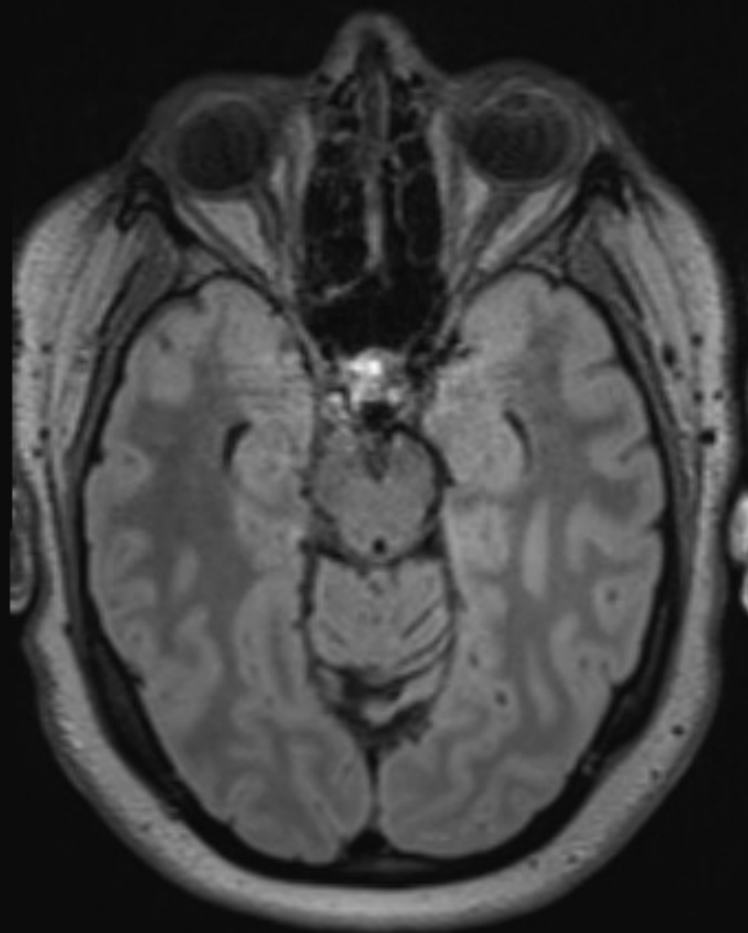

Supplement: Supplementary file 1 — Figure S1. Brain MRI of the more severely affected sister (Patient II). (A) Coronal and (B) axial T2‐weighted Fluid Attenuated Inversion Recovery (FLAIR) sequence without any structural (epileptogenic) abnormalities. [file ACN3-6-2317-s001.pdf]
